# Effect of sulfate on low-temperature anaerobic digestion

**DOI:** 10.3389/fmicb.2014.00376

**Published:** 2014-07-24

**Authors:** Pádhraig Madden, Abdul M. Al-Raei, Anne M. Enright, Fabio A. Chinalia, Dirk de Beer, Vincent O'Flaherty, Gavin Collins

**Affiliations:** ^1^Microbiology, School of Natural Sciences, National University of Ireland GalwayGalway, Ireland; ^2^Department of Biogeochemistry, Max Planck Institute for Marine MicrobiologyBremen, Germany; ^3^Centre for Resource Management and Efficiency, School of Applied Science, Cranfield UniversityBedfordshire, UK; ^4^Ryan Institute for Environmental, Marine and Energy Research, National University of Ireland GalwayGalway, Ireland; ^5^Infrastructure and Environment, School of Engineering, University of GlasgowUK

**Keywords:** biogas, low-temperature anaerobic digestion, sulfate, sulfide, methane, methanogenesis, wastewater

## Abstract

The effect of sulfate addition on the stability of, and microbial community behavior in, low-temperature anaerobic expanded granular sludge bed-based bioreactors was investigated at 15°C. Efficient bioreactor performance was observed, with chemical oxygen demand (COD) removal efficiencies of >90%, and a mean SO^2−^_4_ removal rate of 98.3%. *In situ* methanogensis appeared unaffected at a COD: SO^2−^_4_ influent ratio of 8:1, and subsequently of 3:1, and was impacted marginally only when the COD: SO^2−^_4_ ratio was 1:2. Specific methanogenic activity assays indicated a complex set of interactions between sulfate-reducing bacteria (SRB), methanogens and homoacetogenic bacteria. SO^2−^_4_ addition resulted in predominantly acetoclastic, rather than hydrogenotrophic, methanogenesis until >600 days of SO^2−^_4_-influenced bioreactor operation. Temporal microbial community development was monitored by denaturation gradient gel electrophoresis (DGGE) of 16S rRNA genes. Fluorescence *in situ* hybridizations (FISH), qPCR and microsensor analysis were combined to investigate the distribution of microbial groups, and particularly SRB and methanogens, along the structure of granular biofilms. qPCR data indicated that sulfidogenic genes were present in methanogenic and sulfidogenic biofilms, indicating the potential for sulfate reduction even in bioreactors not exposed to SO^2−^_4_. Although the architecture of methanogenic and sulfidogenic granules was similar, indicating the presence of SRB even in methanogenic systems, FISH with rRNA targets found that the SRB were more abundant in the sulfidogenic biofilms. *Methanosaeta* species were the predominant, keystone members of the archaeal community, with the complete absence of the *Methanosarcina* species in the experimental bioreactor by trial conclusion. Microsensor data suggested the ordered distribution of sulfate reduction and sulfide accumulation, even in methanogenic granules.

## Introduction

The application of anaerobic digestion (AD) is an efficient approach for the treatment of high-strength organic wastewater (Yu et al., 2005a). AD does not require costly aeration and is thus considered more sustainable than aerobic systems (Rittmann and McCarty, 2001). Moreover, anaerobic systems generate re-usable biogases and significantly less nuisance, excess sludge (Yu et al., 2005a). Furthermore, low-temperature (>20°C) AD (LtAD) has been demonstrated as a feasible approach for wastewater treatment (e.g., Connaughton et al., 2006; Akila and Chandra, 2007; Enright et al., 2009), allowing for further efficiencies by eliminating the need to heat AD bioreactors, and opening AD to new areas of environmental management, including for the digestion of raw sewage in temperate climates (Lew et al., 2004).

AD has also been applied in the treatment of sulfate-rich wastewaters. Many industrial processes that use sulfuric acid (e.g., fermentation or seafood processing); or reduced sulfur compounds i.e., sulfide (e.g., in tanneries, kraft pulping), sulfite (e.g., sulfite pulping), thiosulfate (e.g., fixing of photographs) or dithionite (e.g., pulp bleaching) generate sulfate-contaminated wastewaters (Hulshoff Pol et al., 1998).

In the absence of oxygen, sulfate-reducing bacteria (SRB) use sulfate as electron acceptor in the oxidation of an energy substrate with the production of hydrogen sulfide (H_2_S; Boshoff et al., 2004). Sulfate-rich wastewaters stimulate SRB growth, which can out-compete methanogens for substrates (e.g., H_2_, CO_2_ and acetate) in anaerobic environments (Kristjanson et al., 1982; Schonheit et al., 1982), such as in AD bioreactors. Furthermore, SRB consume hydrogen below a minimum threshold for hydrogen metabolism by methanogens (Lovley, 1985; Lovley and Ferry, 1985). Thus, sulfate reduction can impair methane production in wastewater treatment systems.

A particular problem arising from SRB activity is H_2_S production (Koschorreck, 2008). H_2_S is a potentially toxic gas, which is an industrial and municipal nuisance due to its flammability, as well as the corrosive effect on steel and concrete owing to sulfuric acid generation. Additionally, there is a negative effect on microbial cells due to the precipitation of essential trace metals as metal sulfides. Moreover—though depending on the charge of the sulfide ion—H_2_S can have a toxic effect on cellular mechanics as neutrally-charged H_2_S can be transported across the cell membrane, thus increasing the potential for toxicity (Tursman and Cork, 1989; Moosa and Harrison, 2006).

The impact of sulfate ions on AD has been investigated using specific methanogenic activity (SMA) assays and toxicity assays. For instance, O'Flaherty et al. (1998a,b) found competition between SRB and methanogens for available substrates, as well as impaired methanogenesis due to sulfide toxicity, which resulted in reduced methane production. In any case, sulfide toxicity is unlikely to be de-coupled from competition between SRB and methanogens, and due to their more favorable growth and thermodynamic properties, SRB are considered to out-compete other anaerobes in the presence of excess sulfate. O'Flaherty and Colleran (1999), O'Flaherty et al. (1999), and Pender et al. (2004) showed that acetoclastic methanogenesis was the most susceptible reaction to H_2_S inhibition. The outcome of the competition is important, as it determines the relative concentrations of biogas sulfide and methane (Hulshoff Pol et al., 1998). The chemical oxygen demand (COD): SO^2−^_4_ ratio in the influent wastewater is also important. For wastewater with a COD: SO^2−^_4_ ratio of 0.66, there is theoretically sufficient sulfate available to SRB to completely remove the organic matter (Rinzema and Lettinga, 1988); however, for lower COD: SO^2^_4_ ratios, the organic matter is insufficient for complete SO^2−^_4_ reduction. Similarly, for wastewaters with higher COD: SO^2−^_4_ ratios, complete removal of organic matter can only be achieved with concomitant methanogenesis and sulfidogenesis (Omil et al., 1997).

In this study, expanded granular sludge bed (EGSB) bioreactors were used to investigate SRB activity in low-temperature anaerobic digesters. The impact of sulfate contamination on methanogenesis, as well as on community structure, and the distribution and abundance of SRB functional genes, was assessed at different COD: SO^2−^_4_ ratios.

## Materials and methods

### Source of biomass

Anaerobic sludge was obtained from a full-scale, granular biomass nursery plant operated at 30°C in the Netherlands (Paques B.V.). The sludge consisted of well-settling, green-gray granules (Ø, 0.5–3 mm) with a volatile suspended solids (VSS) content of 73 g l^−1^.

### Bioreactor design and operation

**Figure 1:**
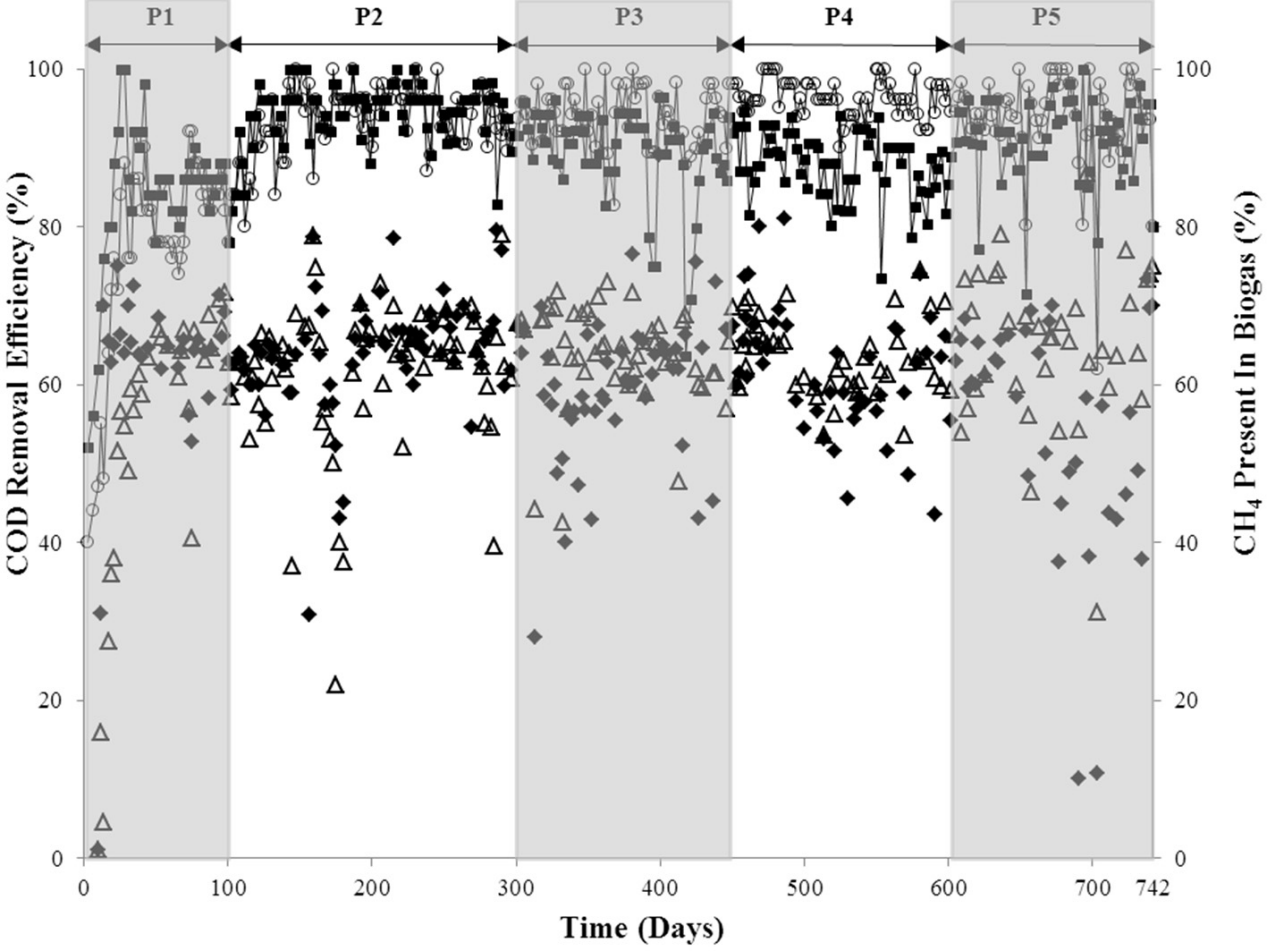
**COD removal efficiency of R1 (◦) and R2 (■); and biogas methane concentrations of R1 (△) and R2 (♦) over each of the five periods (P1–P5)**.

Two glass, laboratory-scale (3.8 l active volume), hybrid, expanded granular sludge bed-anaerobic filter (EGSB-AF) bioreactors (R1 and R2), which were of the same design as described by McHugh et al. (2004), were used for this study. A total mass of 73 g VSS of the sludge was used to seed each bioreactor. R1 and R2 were each fed a synthetic wastewater (pH 7.25 ± 0.2) consisting of acetate, propionate, butyrate, ethanol and glucose in the COD ratio of 1:1:1:1:1, to a total of 5 g COD l^−1^. The influent was buffered with NaHCO_3_ (8 g l^−1^) and fortified, as described by Shelton and Tiedje (1984), with macro- (10 ml l^−1^) and micro- (1 ml l^−1^) nutrients. The trial was accordingly divided into five experimental periods (P1-P5), as indicated in Table 1 and Figure 1, where operating parameters of all bioreactors are outlined.

**Table 1:** **Operational periods, and associated parameters, of R1+2**.

**Period**		**P1**	**P2**	**P3**	**P4**	**P5**
Days		0–95	96–299	300–457	458–604	605–742
Bioreactor temperature^a^		15	15	15	15	15
Influent COD^b^		5	5	5	5	5
Upflow velocity^c^		5	5	5	5	5
HRT (h)		36	24	24	24	24
OLR^d^		0.288	0.433	0.433	0.433	0.433
OLR^e^		3.32	5	5	5	5
*VLR*^f^		0.66	1.0	1.0	1.0	1.0
% CH_4_ in Biogas	*R1*	52.45 (0.65)	62 (0.1)	64 (0.1)	63.2 (0.1)	64.5 (0.3)
	*R2*	61.5 (0.5)	64 (0.1)	60 (0.15)	62.1 (0.15)	53.7 (0.5)
% COD Removal	*R1*	76.42 (2.13)	92.92 (0.5)	94.15 (0.4)	96.15 (0.3)	93.85 (0.8)
	*R2*	83.52 (1.62)	94.04 (0.46)	89.61 (0.74)	87.71 (0.52)	91.15 (0.7)
Influent SO^2−^^g^_4_	*R1*	–	–	–	–	–
Influent SO^2−^^g^_4_	*R2*	–	–	0.625	1.66	10
Influent SO^2−^^h^_4_	*R2*	–	–	0.0065	0.017	0.1
Effluent SO^2−^^i^_4_	*R1*	–	–	–	–	–
Effluent SO^2−^^i^_4_	*R2*	–	–	10.37 (1.95)	291.52 (27.0)	3776.29 (126.24)
Effluent H_2_S^i^	*R1*	–	–	–	–	–
Effluent H_2_S^h^	*R2*	–	–	0.69 (0.18)	176.27 (13.44)	116.49 (4.86)
SO^4^ Rate^i^	*R2*			46.71 (0.02)	201.56 (0.49)	862.06 (2.21)
H2S Rate^k^	*R2*	–	–	0.052 (0.001)	25.96 (0.25)	16.04 (0.09)

Standard errors (standard deviation/vn, where n is the number of days in a given period) are presented in parentheses.

a Degrees Celsius.

b g l^−1^.

c m h^−1^.

d OLR expressed as kg COD kg (VSS)^−1^ d^−1^.

e OLR expressed as kg COD m^−3^ d^−1^.

f m3_wastewater_ m^−3^_reactor_ d^−1^.

g g l^−1^.

h M l^−1^.

i mg l^−1^.

j Average g sulfate reduced Kg (VSS)^−1^ d^−1^.

k Average g sulfide produced kg (VSS)^−1^ d^−1^.

### Routine analytical techniques

Samples of bioreactor effluent and biogas were routinely sampled to determine concentrations of COD and methane, respectively, according to Standard Methods (APHA, 1998). Residual sulfate concentrations and sulfide production were routinely (average, 4 times per week) determined using a colorimetric spectrophotometer (HACH, Colorado, USA).

### Determination of sulfidogenic rates

The rate of R2 sulfate reduction (SRR) was calculated for P3, P4, and P5 as a function of the VSS content (kg) of the bioreactor, which was based on the measured VSS concentration (g/100 ml) of typical granules sampled from R2 on days 304, 449, 605, and at the conclusion of the trial. The rate of R2 sulfide production (SPR) was calculated similarly.

### DNA extraction and PCR-amplification

A DNA extraction protocol (Griffiths et al., 2000) was applied for the recovery of total genomic DNA from sludge granules (0.5 g) sampled from the sludge bed of R1 and R2 on days 0 (seed sludge), 136 (P2), 304 (P3), 356 (P3), 455 (P3), 515 (P4), 602 (P4), and 742 (P5). 16S rRNA gene fragments were amplified using the *Archaea*-specific primer set A751F and UA1204R (Baker et al., 2003), and the *Bacteria*-specific primer set EB341F and UN517R (Muyzer et al., 1993). PCR-amplification of the archaeal and bacterial 16S rRNA genes was performed as described in detail by Madden et al. (2010). Dissimilatory sulfite reductase ß-subunit (dsrB) gene fragments were amplified using dsrB-specific primers DSRp2060F (Geets et al., 2006) and DSR4R (Wagner et al., 1998) to yield a dsrB gene fragment of ~350 bp. A 40-base-pair GC-clamp (Muyzer et al., 1993) had been added to the 5′-end of A751F, EB341F, and DSRp2060F. PCR assays, including no-template controls, using dsrB primers were performed in 50-μl reactions containing: ~200 ng of template DNA, 12.5 pmol of each primer, 1.5 mmol MgCl_2_, 5 μl 1× NH_4_ reaction buffer [16 mM (NH_4_)_2_SO_4_, 67 mM Tris-HCl (pH 8.8 at 25°C), 0.01% Tween-20], 500 nmol dNTP (125 nmol of each of dATP, dCTP, dGTP, and dTTP) and 2 U *Taq* DNA polymerase (Bioline, London, UK). The dsrB PCR conditions were: denaturation at 94°C for 3 min; 9 cycles of denaturation at 94°C for 30 s, annealing of primers at 60°C for 30 s (−1°C at each cycle) and extension at 72°C for 45 s; this was then followed by 24 cycles of denaturation at 94°C for 30 s, annealing of primers at 55°C for 30 s and extension at 45°C for 45 s. Final extension at 72°C was for 7 min.

### DGGE and analysis of 16S rRNA and dsrB gene fragments

Community-based patterns were generated by denaturing gradient gel electrophoresis (DGGE) of archaeal and bacterial 16S rRNA, and dsrB, gene PCR products. Polyacrylamide gels (8% [w/v]; thickness, 1 mm) with a denaturing gradient consisting of 30–60% urea-formamide for archaeal and dsrB samples, or 30–70% urea-formamide for bacterial samples, were used. DGGE was performed, and bands were excised and re-amplified, as described in detail by Madden et al. (2010). PCR amplicons from excised bands were sequenced by MWG (UK) using Sanger sequencing technology. Gene sequences from this study were deposited in *Genbank* under accession numbers FJ535442-FJ535447 for *Archaea*, FJ535448-FJ535456 for *Bacteria* and FJ535457-FJ535466 for dsrB genes (Table 2), with the following nomenclature and generic prefixes: ARC-PM1, ARC-PM2 and ARC-PM4 to ARC-PM7 for archaeal sequences; B1-PM to B6-PM and B10-PM to B12-PM for bacterial sequences; and SRB1-PM to SRB-PM10 for dsrB sequences (Table 2). DGGE data were analyzed as described by Madden et al. (2010).

**Table 2:** **Origin and closest relatives of excised DGGE bands**.

**DGGE Band**	**Genbank accession**	**Biomass**	**Closest relative from blastn** (***accession number)***	**Similarity** (**%)**
**A. ORIGIN AND CLOSEST RELATIVES OF EXCISED ARCHAEAL DGGE BANDS**				
1	FJ535442	R2 day 304	Uncultured archaeon gene	100
2	FJ535443	R1 day 304	Uncultured archaeon gene	99
4	FJ535444	R1 day 304	Uncultured archaeon clone TDC-AR4	98
5	FJ535445	R1 day 304	Uncultured *Methanosaeta* species	99
6	FJ535446	R1 day 742	Uncultured archaeon clone SCA49	98
7	FJ535447	R2 day 742	Uncultured archaeon clone 06-02-208	98
**B. ORIGIN AND CLOSEST RELATIVES OF EXCISED BACTERIAL DGGE BANDS**				
1	FJ535448	R2 day 304	Uncultured delta proteobacterium clone 1R2U70	100
2	FJ535449	R1 day 304	Uncultured bacterium clone FLSED43	94
3	FJ535450	R2 day 304	Uncultured bacterium clone FLSED43	92
4	FJ535451	R1 day 515	Uncultured bacterium clone FLSED5	95
5	FJ535452	R1 day 136	Uncultured bacterium clone 32g06	99
6	FJ535453	R1 day 742	Uncultured delta proteobacterium clone 1R2U28	98
10	FJ535454	R2 day 515	Uncultured delta proteobacterium clone 1R2U70	100
11	FJ535455	R2 day 742	*Chlorobium limicola* DSM 245, complete genome	99
12	FJ535456	R2 day 742	*Chlorobaculum tepidum* partial 16S rRNA gene	97
**C. ORIGIN AND CLOSEST RELATIVES OF EXCISED SRB DGGE BANDS**				
1	FJ535457	R2 day 304	Uncultured bacterium clone NTUA-5A-DSR2 dsrA and dsrB genes, partial cds	99
2	FJ535458	Day 0	Desulfobacterium autotrophicum partial dsrA and dsrB genes	87
3	FJ535459	R2 day 136	Uncultured sulfate-reducing bacterium isolate DGGE gel band 08 dsrB gene, partial cds	82
4	FJ535460	R1 day 136	Uncultured sulfate-reducing bacterium isolate DGGE gel band 08 dsrB gene, partial cds	82
5	FJ535461	R1 day 136	Uncultured sulfate-reducing bacterium clone GranDSR8 dsrA and dsrB genes, partial cds	98
6	FJ535462	R1 day 136	Uncultured sulfate-reducing bacterium isolate DGGE gel band 08 dsrB gene, partial cds	81
7	FJ535463	R2 day 136	Uncultured bacterium clone NTUA-5A-DSR2 dsrA and dsrB genes, partial cds	99
8	FJ535464	R1 day 304	Uncultured bacterium clone NTUA-5A-DSR2 dsrA and dsrB genes, partial cds	99
9	FJ535465	R2 day 515	Uncultured bacterium clone NTUA-5A-DSR2 dsrA and dsrB genes, partial cds	99
10	FJ535466	R1 day 304	*Desulfomicrobium* sp. ADR28 partial dsrA gene and partial dsrB gene, strain ADR28	95

### Real-time PCR analysis

Quantitative, real-time PCR assays were performed using a LightCycler 480 (Roche, Mannheim, Germany). Four methanogenic primer and probe sets (Yu et al., 2005b; Lee et al., 2009), specific for two orders (*Methanomicrobiales* and *Methanobacteriales*) and two families (*Methanosaetaceae* and *Methanosarcinaceae*) were used (Table 3). One bacterial primer and probe set was also used (Yu et al., 2005b; Lee et al., 2009). Archaeal and bacterial reaction mixtures were prepared as described by O'Reilly et al. (2010).

**Table 3:** **Characteristics of the real-time PCR primer and probe sets used in this study**.

**Set name/target group**	**Sequence (5′—3′)^a^**	**Representative strains^b^**
**MBT-set/Methanobacteriales^c^**	F: CGWAGGGAAGCTGTTAAGT	Methanobacterium thermoautotrophicum (DSM1053)
	T: AGCACCACAACGCGTGGA	Methanobrevibacter arboriphilicus (DSM 1536)
	R: TACCGTCGTCCACTCCTT	
**MMB-set/Methanomicrobiales^c^**	F: ATCGRTACGGGTTGTGGG	Methanocorpusculum parvum (DSM 3823)
	T: TYCGACAGTGAGGRACGAAAGCTG	Methanomicrobium mobile (DSM 1539)
	R: CACCTAACGCRCATHGTTTAC	Methanospirillum hungatei (DSM 864)
**Mst-set/Methanosaetaceae^c^**	F: GAAACCGYGATAAGGGGA	Methanosaeta concilii (DSM 2139)
	T: TTAGCAAGGGCCGGGCAA	Methanosaeta thermoacetophila (DSM6194)
	R: TAGCGARCATCGTTTACG	
**Msc-set/Methanosarcinaceae^c^**	F: TAATCCTYGARGGACCACCA	Methanosarcina acetivorans (DSM 2834)
	T: ACGGCAAGGGACGAAAGCTAGG	Methanosarcina barkeri (DSM 800)
	R: CCTACGGCACCRACMAC	Methanosarcina mazei (DSM 3647)
**BAC set/Bacteria^c^**	F: ACTCCTACGGGAGGCAG	Escherichia Coli K12 (DSM 498)
	T: TGCCAGCAGCCGCGGTAATAC	
	R: GACTACCAGGGTATCTAATCC	
**DsrB-set^d^**	F: CAACATCGTYCAYACCCAGGG	Desulfovibrio longus (DSM 6739^T^)
	R: GTGTAGCAGTTACCGCA	

a F, T, and R indicate forward primer, TaqMan probe, and reverse primer, respectively.

b Culture collection numbers are in parentheses.

c Yu et al. (2005a), Lee et al. (2009).

d Geets et al. (2006), Wagner et al. (1998).

The dsrB reaction mixtures were prepared using the LightCycler 480 SYBR Green I Master kit (Roche): 3 μl of PCR-grade water, 10 μl of SYBR green reaction solution (final conc. 200 nM), 1 μl of each primer (final conc. 500 nM), and 5 μl of DNA template. The amplification consisted of 45 cycles, with 1 cycle of denaturation (95°C for 40 s), annealing (55°C for 40 s), and elongation (72°C for 1 min).

Quantitative standard curves were constructed using the standard plasmids containing the full-length 16S rRNA gene sequences from the representative strains of the target methanogenic and bacterial groups as previously described (Yu et al., 2005b; Lee et al., 2009). *Desulfovibrio longus* 6739^T^ (Magot et al., 1992), grown up in desulfovibrio medium no. 63 (DSMZ), was used as a source of dsrB gene sequences. Standard curves and analysis were performed as described by O'Reilly et al. (2010).

### Specific methanogenic activity (SMA) assays

SMA assays were performed as described by Colleran et al. (1992) and Coates et al. (1996) using the seed inoculum and granular biomass samples recovered from the bioreactors at days 449, 605 and at the conclusion of the experiment (Table 4). The substrates tested, and the concentrations used, were acetate (30 mM), butyrate (15 mM), propionate (30 mM), ethanol (30 mM), and H_2_/CO_2_ (80:20 v/v), as described in greater detail by Collins et al. (2003). All tests were performed with and without the addition of sulfate (Table 4).

**Table 4:** **SMA data for seed sludge and temporal biomass from R4 and R5**.

**Bioreactor**	**Test temp (°C)**	**SO_4_ +/−**	**Test day**	**Acetate**	**H_2_/CO_2_**	**Propionate**
Inoculum	15	−	0	21.5 (0.9)	73.3 (18.9)	11.4 (0.5)
Inoculum	37	−	0	72.9 (4.9)	118.6 (7.6)	96.8 (3)
R4	15	−	449	31 (0)	147.5 (3.2)	91.9 (3.2)
R4	15	+	449	24.4 (0.2)	63.4 (1.2)	51.7 (2.9)
R4	37	−	449	346.9 (5.8)	523.3 (24.2)	334.3 (2.6)
R4	37	+	449	281.4 (14.5)	531.8 (0.6)	104.5 (2.9)
R5	15	−	449	95.5 (2.6)	30.5 (0.03)	4.5 (2.2)
R5	15	+	449	60 (8.5)	28.6 (3.6)	8.6 (3)
R5	37	−	449	266.1 (24)	279.7 (25)	26.7 (2.5)
R5	37	+	449	323.7 (11.3)	405.3 (2.7)	6.3 (0.2)
R4	15	−	605	73.2 (2.4)	180.4 (33.5)	87.7 (1.7)
R4	15	+	605	41.6 (3.5)	63.1 (1.5)	91.5 (10.7)
R5	15	−	605	186.4 (64.2)	35.4 (5.1)	3.8 (0.1)
R5	15	+	605	58.1 (2.3)	57.6 (9.7)	2.5 (0.1)
R4	15	−	742	69.5 (4.3)	131.7 (3.1)	76.9 (6.7)
R4	15	+	742	34.8 (0.3)	57.5 (0.3)	19.2 (1)
R4	37	−	742	107 (3.5)	201.5 (13.5)	154.6 (34.7)
R4	37	+	742	57.7 (2.5)	154.1 (2.3)	89.1 (4.7)
R5	15		742	42.5 (3)	67.4 (2.2)	1.6 (0.1)
R5	15	+	742	37.4 (0.8)	48.6 (0.5)	17.9 (1.9)
R5	37	−	742	461.2 (16.5)	184.2 (5.6)	2.8 (1.6)
R5	37	+	742	234.1 (7.5)	112.8 (1.9)	9.7 (1.1)

Values are expressed in ml CH_4_ g^−1^ VSS^−1^ day^−^ and are the mean of triplicates. Standard errors (standard deviation/vn, where n = 3) are in parentheses.

### Microsensor measurements

Microsensor analysis was applied to study granules from both bioreactors on day 625 and at the conclusion of the trial (day 742). Single granules were stacked on top of each other in glass capillary tubes (Ø, 10 mm; height, 180 mm), which were sealed at the base. The stack of granules was then completely immersed in anaerobic medium. Anaerobic conditions were maintained by continuous bubbling of the mixture with argon gas, and the apparatus was placed in a 15°C water bath to simulate, as closely as possible, the distribution, and physico-chemical conditions, of anaerobic granules in the bioreactors. After incubation for 24 h, microprofiles were recorded by penetrating the granules with microsensors in increments of 20 or 50 μm and at time intervals of 10 or 20 s. A dissection microscope was used to monitor complete microsensor penetration into each individual granule.

#### Hydrogen sulfide microsensors

Sulfide concentration profiles were measured with H_2_S microsensors (Jeroschewski et al., 1996; Kuhl et al., 1998) with a tip diameter of 30 μm and a 90% response time of <0.5 s. The microsensors were calibrated in accordance with the colorimetric methylene blue method (Fonselius et al., 1999). The concentration of total dissolved sulfide (H_2_S + HS^−^ + S^2−^) in the dilution series was determined by spectrophotometry (Cline, 1969). Calibration was performed in a medium of the same pH as the granules and incubation medium; therefore no pH correction was necessary. The sensor showed a linear response to H_2_S concentrations of up to 1000 μM and the detection limit of the microsensors was 1 μM total sulfide.

#### Sulfate microsensors

The sulfate microsensor used was a liquid-ion exchange (LIX) microelectrode. The filling electrolyte used was 300 mM KCl. The filling solution was degassed under vacuum and filtered through a 0.2-μm-pore-size Millipore membrane. The silanized capillaries were filled with electrolyte by using a plastic syringe drawn in a flame to a 0.1-mm tip; applying pressure from the back pushed out the air pocket that typically was left in the tip. Then, under microscopic inspection, the tips were dipped in LIX and suction was applied until a membrane with a thickness of 300 μm was introduced. The capillary was left for at least 2 h, during which the tetrahydrofuran evaporated and a solid ion-selective membrane was a formed in the tip.

#### pH Microsensors

The pH sensor used was a LIX microelectrode. pH sensors were constructed from raw glass capillaries following the procedure of de Beer et al. (1997) described for nitrite microsensors.

### Granule fixation, sectioning and fluorescence *in situ* hybridization

Granules were fixed by overnight incubation in paraformaldehyde [4% (w/v) in 1× phosphate-buffered saline (PBS)] at 4°C. After washing three times in 1× PBS, fixed granules were incubated in an OCT freezing medium (Sakura Finetek USA, Torrance, Calif.) at 4°C overnight. Embedded granules were then sectioned and prepared for hybridizations as described by Sekiguchi et al. (1999).

The protocols described by Sekiguchi et al. (1999) and Schramm et al. (1998) were used for FISH experiments. Probes were synthesized and labeled with a hydrophilic sulfoindocyanide dye (Cy3 or Cy5) by Interactiva GmbH (Ulm, Germany) (Table 5). Microscopy was with a Nikon Y-FL epifluorescence microscope and Nikon E300. All images were captured using a Qi-camera and QImaging software (QImaging, BC, Canada).

**Table 5:** **Oligonucleotide probes used for PCR and FISH analysis**.

**Probe**	**Position^a^**	**Sequence (5′→3′)**	**Target (reference)**	**Formamide (%)^b^**	**NaCl (mM)^c^**
A751F^d^^,^^e^	–	CCGACGGTGAGRGRYGAA	Archaea (Baker et al., 2003)	–	–
UA1204R^e^	–	TTMGGGGCATRCIKACCT	Archaea (Baker et al., 2003)	–	–
EB341F^d^^,^^e^	–	CCTACGGGAGGCAGCAG	Bacteria (Muyzer et al., 1993)	–	–
UN517R^e^	–	ATTACCGCGGCTGCTGG	Bacteria (Muyzer et al., 1993)	–	–
DSR4R^d^^,^^e^	–	GTGTAGCAGTTACCGCA	dsrB Gene (Wagner et al., 1998)	–	–
DSRp2060F^e^	–	CAACATCGT(CT)CA(CT)ACCCAGGG	dsrB Gene (Geets et al., 2006)	–	–
Eub338	338–355	GCTGCCTCCCGTAGGAGT	Bacteria (Amann et al., 1990)	20	225
Arc915	915–934	GTGCTCCCCCGCCAATTCCT	Archaea (Stahl and Amann, 1991)	40	–
SRB385	385–402	CGGCGTCGCTGCGTCAGG	Most desulfovibrionales (Amann et al., 1992)	35	80
DBB660	660–679	GAATTCCACTTTCCCCTCTG	Desulfobulbus (Devereux et al., 1992)	60	15.6
NON338	338–355	ACTCCTACGGGAGGCAGC	None (Wallner et al., 1993)	–	–

a Position in the 16S rRNA of E. Coli (Brosius et al., 1981).

b Formamide concentration in hybridization buffer.

c Sodium chloride concentration in washing buffer.

d These primers had a 40 base pair GC-clamp at the 5′ end.

e Probes not used for FISH.

## Results

### Bioreactor performance and response to sulfate addition

A start-up period of ~20–25 days was observed, after which, the COD removal efficiency of both bioreactors was 80–90% (P1; Figure 1). The shortened HRT (from 36 to 24 h) in P2 resulted in improved COD removal (P2; Figure 1). On day 300, sulfate was added to the influent of R2 at a COD:SO^2−^_4_ ratio of 8:1 but no difference in the performance of R1 and R2 was detected until day 392, and again at day 418, when reduced R2 COD removal efficiency (to 75 and 64%) was observed (P3; Figure 1). Nonetheless, after a recovery period (of 7 days in both examples), R2 COD removal efficiency returned to ~90%. The COD: SO^2−^_4_ ratio was decreased to 3:1 during P4, which resulted in reduced R2 COD removal. On average, R1 performed better than R2 during P4 (P4; Figure 1). Upon increasing the SO^2−^_4_ concentration for P5 (COD: SO^2−^_4_ ratio of 1:2), the average COD removal efficiency for R2 increased to 91%. This was comparable to R1, with an average COD removal efficiency of 94% (P5; Figure 1). With the exception of during P1, the R1 biogas methane concentration was consistently at 62–64% (average value each period). The concentration of R2 biogas methane, on the other hand, decreased during the final period to 54% (Figure 1).

### Sulfate reduction and sulfide production in bioreactor effluent

Throughout P3 (Table 1), an average of 98.3% of R2 influent sulfate was removed, with an average dissolved effluent sulfide concentration of 0.69 mg l^−1^.

**Figure 2:**
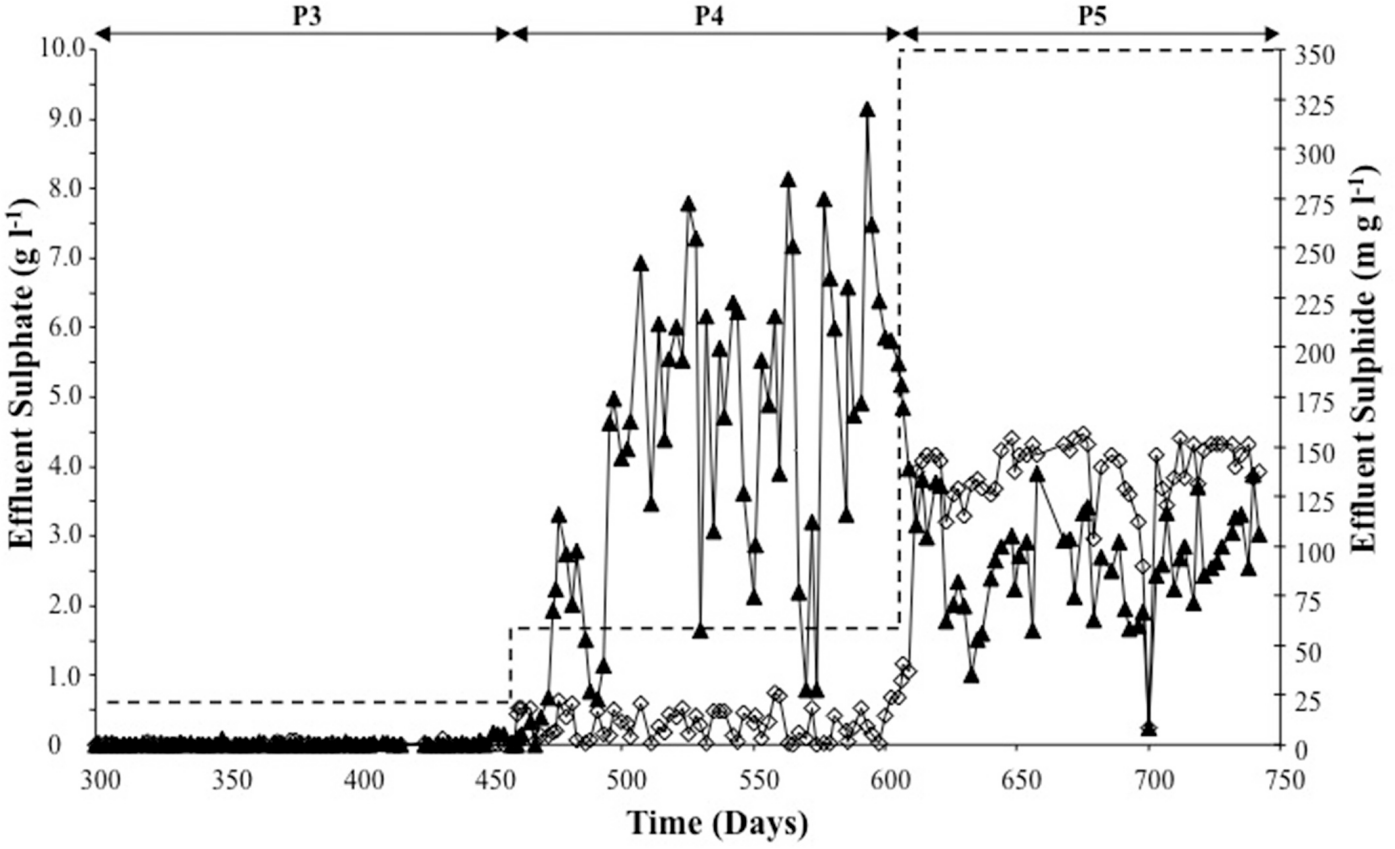
**Sulfate reduction (▲) and sulfide production (◊) determined from analysis of R2 effluent for P3–P5 (during sulfate supply to R2). Dashed line represents R2 sulfate influent for each of the periods P3–P5**.

On day 458 (beginning of P4), influent sulfate dosing was increased from 625 mg l^−1^ to 1660 mg l^−1^. The sulfate removal efficiency during P4 decreased to 82%. The average P4 effluent sulfide concentration was 176 mg l^−1^, which was a 255-fold increase of the P3 average concentration. Indeed, in one instance (day 593), the sulfide concentration was 320 mg l^−1^, which was almost twice the period average (Figure 2).

The average sulfate removal efficiency during P5 decreased (to c. 63%) with increased sulfate dosing to the R2 influent (Figure 2). Despite the increased sulfate dosing during P5, reduced sulfide production (by 50%–116.5 mg l^−1^) was observed (Figure 2).

### Sulfidogenic rates

SRRs and SPRs are presented in Table 1. A steady increase in the SRR was observed from P3 through P5. The SPR increased from P3 to P4, but decreased in P5, which was in line with reduced effluent sulfide concentrations, indicating that the remaining sulfur was present a H_2_S.

### Microbial community development

**Figure 3:**
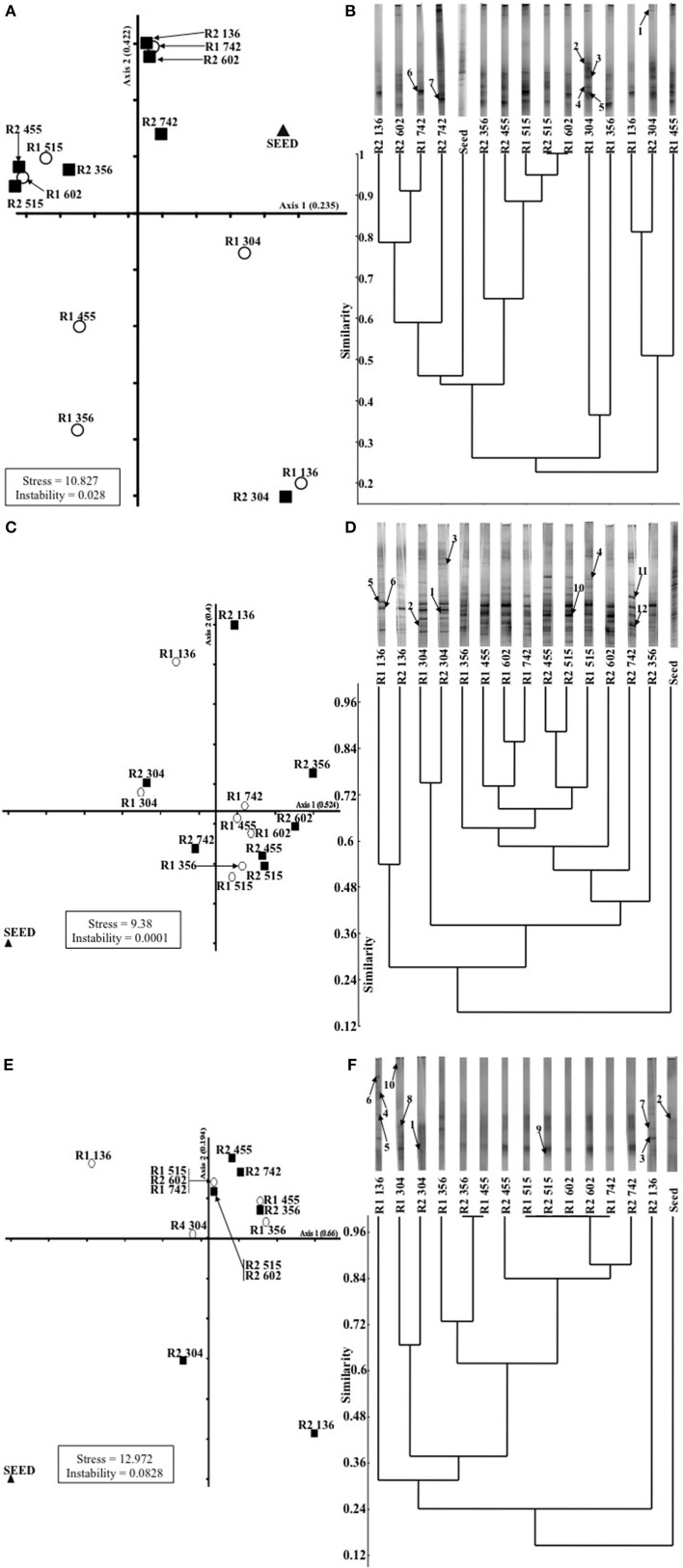
**Non-metric multi-dimensional spacing (NMDS; A,C,E) analysis of (A) archaeal, (C) bacterial and (E) SRB DGGE profiles; and unweighted pair-group methods using arithmetic averages (UPGMA) dendrograms (B,D,F), with associated banding patterns, illustrating temporal analysis of (B) archaeal, (D) bacterial and (F) SRB populations**. Excised bands from DDGE gels (as described in Table 3) are indicated by arrows.

Changes in the microbial populations, as detected by DGGE analysis, were visualized by NMDS analysis because it avoids the assumption of linear relationships among variables and it is reported to be the most generally effective ordination method for ecological community data (McCune and Grace, 2002). Firstly, for the *Archaea*, a migration through all four quadrants for the control bioreactor (R1) occurred (Figure 3). Migration starts with the inoculum in the upper-right quadrant and moves down to the bottom-right quadrant for the next two samples (d 136, P2; and d 304, P3). Community succession is indicated by further movement to the bottom-left quadrant (days 356, 455); the top-left quadrant (days 515, 602); and, finally, at the top-right quadrant (the final sample on day 742), which was relatively close to the inoculum (Figure 3). However, in the case of R2, with the exception of one sample (d 304); only limited movement occurred between the two upper quadrants (Figure 3).

For the bacteria, the plot indicated closely replicated R1 and R2 community structure prior to, and immediately after, the addition of sulfate to R2 influent (Figure 3; days 136, P2; and 304, P3). Based on samples from 56 days after the addition of sulfate, diverged bacterial communities were apparent. No discernable deviation was apparent for the remainder of P3 or during P4 (Figure 3). However, samples from the conclusion of the trial indicated diverged communities during the final period (Figure 3).

Finally, the greatest R1 and R2 similarity was in the plot of temporal dsrB gene fragments (Figure 3). Outside the inoculum, only two samples were outside the top-right quadrant of the plot (Figure 3).

### Specific methanogenic activity (SMA) assays

The SMA of the seed sludge against each of the substrates was higher when tested at 37°C than at 15°C (Table 4). Methanogenic activity was highest against H_2_/CO_2_ at both temperatures. In addition, activity was observed against propionate.

#### SMA assays at 15°C

On day 449 (P3), SMAs were, again, higher at 37°C than at 15°C against each substrate tested. In R1 biomass, methanogenic activity against H_2_, in tests performed without the addition of SO^2−^_4_, was doubled compared to the seed sludge. In fact, the pathway of methane production in R1 appeared to be through H_2_, irrespective of the presence of SO^2−^_4_. Nonetheless, the presence of SO^2−^_4_ in R1 assays did impair methane production (Table 4). In R2, on the other hand, the main route of methane production appeared to be through acetoclastic methanogenesis (Table 4). High activity on acetate, even after c. 150 d with SO^2−^_4_-contaminated influent in R2, points to the maintenance of an active acetoclastic methanogenic community. The presence of SO^2−^_4_ in the assays impaired acetoclastic activity but the presence of SO^2−^_4_ appeared to have little effect on methane production from H_2_/CO_2_. Finally, reduced propionate-degrading activity, compared with the seed sludge, was measured in R2 biomass, with and without SO^2−^_4_ amendment.

Assays on day 605 (P5) indicated that hydrogenotrophic methanogenesis dominated the R1 community. At the same time, the assays indicated further development of the acetoclastic SMA in R2, which was still impaired by SO^2−^_4_ addition in the assays. High activity on propionate was observed in R1 assays, but not in R2 biomass (Table 4).

By day 742, reduced methanogenic activity was observed against acetate in R1 and R2. Methanogenesis in R1 was still dominated by hydrogenotrophy and activity was still impaired with the addition of SO^2−^_4_. However, in R2 biomass, most of the methanogenic activity potential was routed through H_2_, for the first time during the trial (Table 4).

#### SMA assays at 37°C

SMAs were higher at 37°C than at 15°C against each substrate tested in P3 (Day 449), with the exception of SO^2−^_4_ amended R2 assays against propionate (Table 4). R1 assays indicated a H_2_-mediated methanogenic pathway, coupled with prominent propionate degradation. Similarly, R2 assays also indicated a dominant hydrogenotrophic methanogenic community. SO^2−^_4_ impairment of R2 biomass was only observed in propionate-fed assays, whereas acetate- and H_2_/CO_2_–fed SO^2−^_4_-amended assays performed better than the non-amended assays (Table 4).

On day 742 (P5), assays indicated decreased methanogenic activity compared to day 449. Nonetheless, H_2_-mediated methanogenesis appeared to still be the dominant pathway in R1. However, SO^2−^_4_ impairment was observed against each substrate in R1 assays (Table 4). R2 assays in P5 showed that acetoclastic methanogenesis was the main route for methanogenesis compared with day 449 (P3), when hydrogenotrophic methanogenesis appeared dominant. Furthermore, SO^2−^_4_ impairment was observed for two of the three substrates. Contrary to assays performed on day 449, the activity in the R2 SO^2−^_4_ amended, propionate-fed assays was greater than in non-amended assays (Table 4).

### Analysis of microsensor measurements

The SO^2−^_4_ profile of a typical R1 sludge granule (Ø, ~2 mm), sampled on day 742, indicated the internal concentration ranged from ~4.45 mM at the surface to 4.0 mM at the center of the granule. As the microsensor continued through the granule, the sulfate concentration peaked again at 4.45 mM close to the bottom surface. R1 H_2_S profiles indicated concentrations between 2.75 μM at the outer layers and 22 μM at the core. Only a marginal reduction was observed in sulfide production as the microsensor moved through to the bottom surface. The R1 pH microsensor profile indicated only a slight decrease in pH, ranging from 8.52 at the edge to ~8.44 at the center.

**Figure 4:**
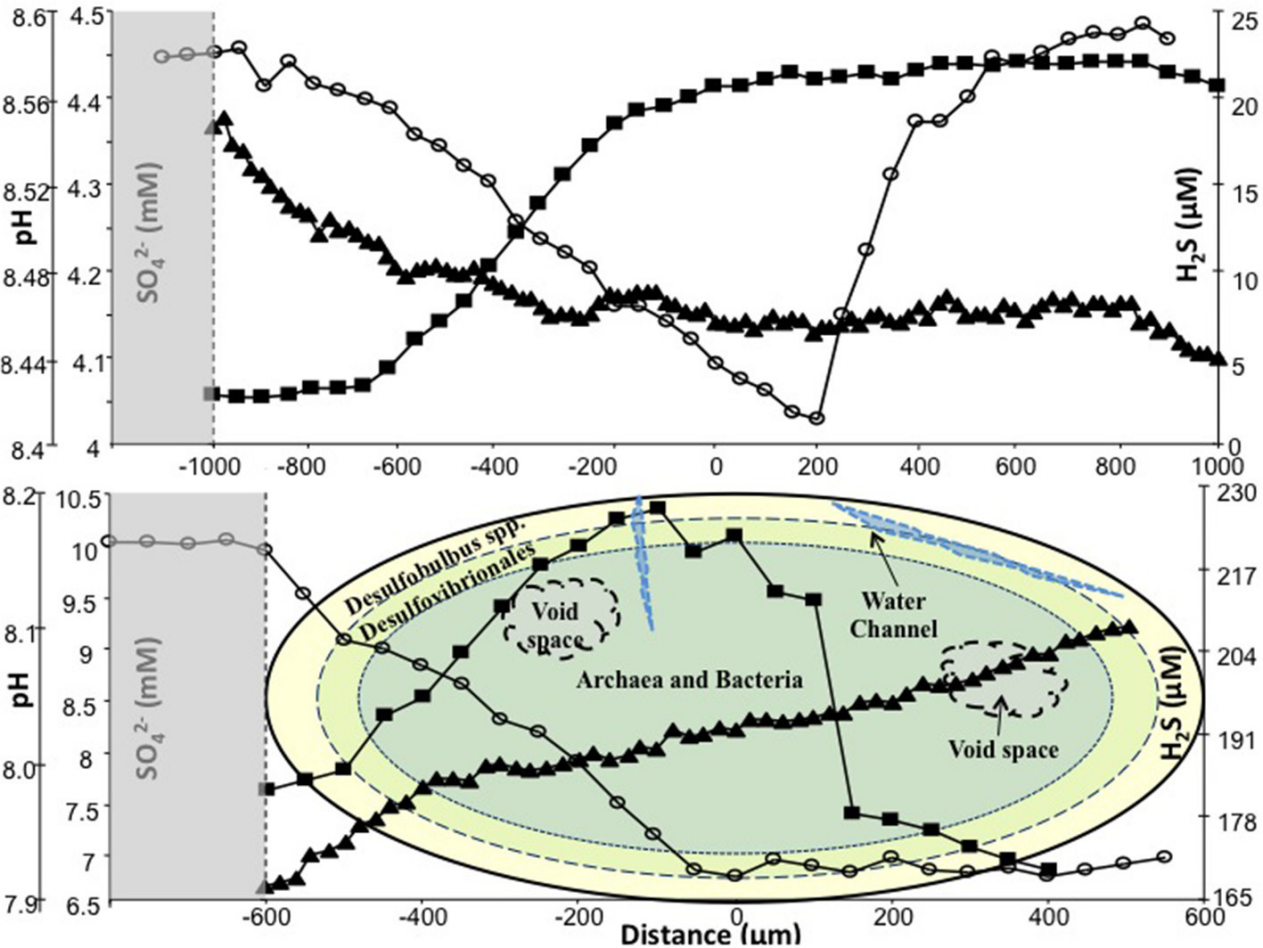
***In situ* SO_4_ (◦), H_2_S (■) and pH (▲) measured on day 742 using microsensors. Top:** typical R1 granule; **bottom:** typical R2 granule, with illustration of microbial trophic zones. Shaded areas represent data from the external environment of the granules. Zero on the *y*-axis represents center of granule.

The SO^2−^_4_ profile from a typical R2 granule (Ø, 1.1 mm; Figure 4), on day 742, indicated that the concentration ranged from 9.87 mM at the edge to 6.71 mM at the center (c. 0 μm). The sulfate concentration was marginally higher (6.9 μM) toward the bottom (+550 μm) surface (Figure 4). The R2 H_2_S profile indicated a sulfide production range of 183 μM at the outer layer to 226 μM at the center (0 μm). The sulfide concentration decreased, to 169.5 μM, as the sensor moved toward the bottom surface, which resulted in an “∩-shaped” profile (Figure 4). The R2 pH profile indicated a gradual, but continuous, increase as the microsensor moved through the granule (Figure 4).

### Fish analysis

FISH revealed a multilayer structure of the granules, where both sulfate (R2) and non-sulfate (R1) granules displayed a similar microbial hierarchical structure. Dense microbial clusters, along with some void spaces, which possibly were water channels in the biofilm, were observed in all granules tested.

**Figure 5:**
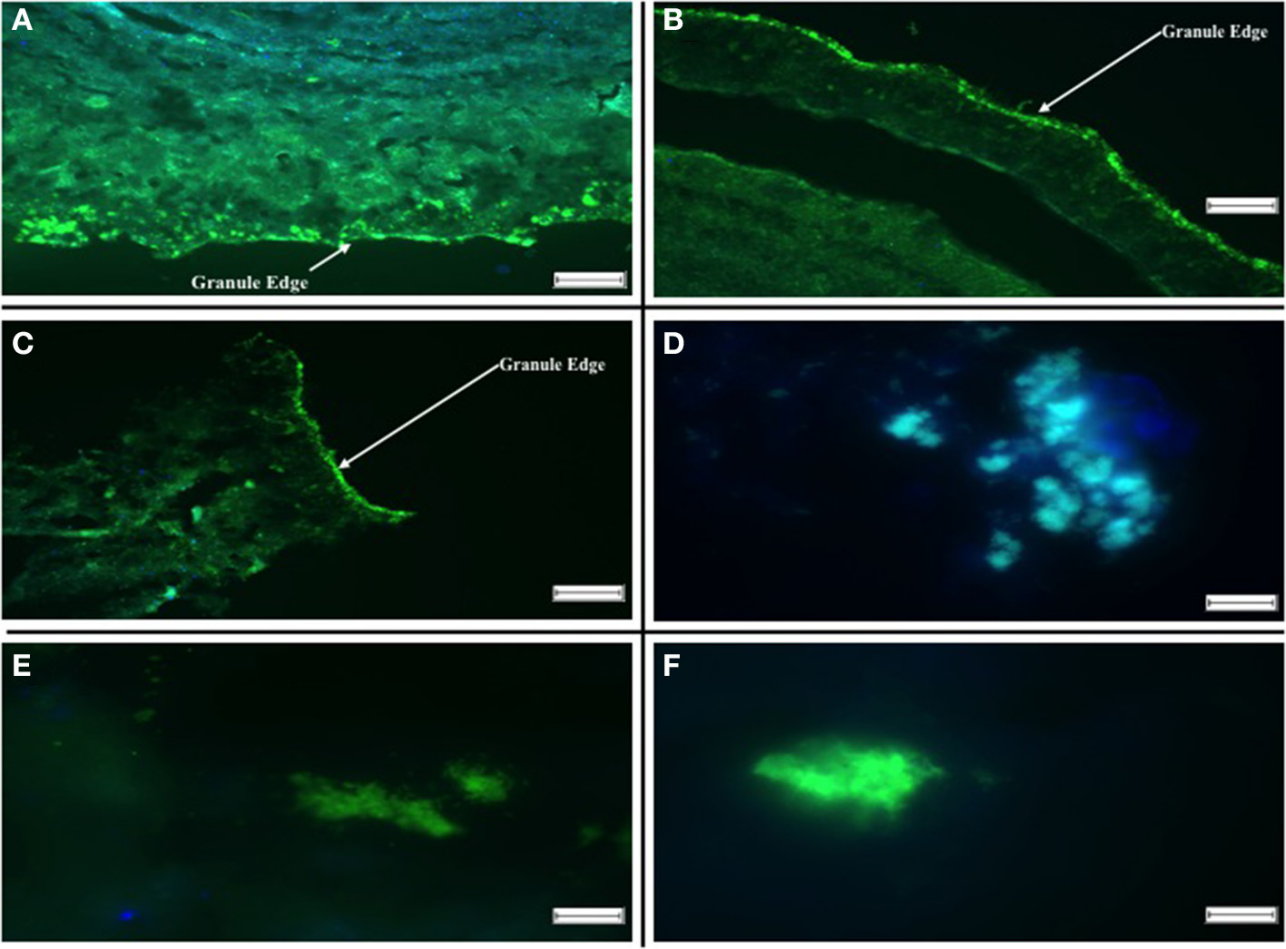
**Fluorescent *in situ* Hybridizations illustrating localization of archaeal and SRB populations in aggregates. (A)** R1 Day 515: Hybridization probes DBB 660 [green] and SRB 385 [blue]. **(B)** R2 Day 515: Hybridization probes DBB 660 [green] and SRB 385 [blue]. **(C)** R1 Day 602: Hybridization probes SRB 385 [green] and ARC 915 [blue]. **(D)** R2 Day 602: Hybridization probes DBB 660 [green] and ARC 915 [blue]. **(E)** R1 Day 742: Hybridization probes DBB 660 [green] and SRB 385 [blue]. **(F)** R2 742: Hybridization probes DBB 660 [green] and SRB 385 [blue]. The scale bar in **(A–C)** is 100 μm, and the arrows indicate the aggregate surface, the scale bar in **(D–F)** is 10 μm.

Using the group-specific oligonucleotide probes DBB 660 and SRB 385, sections of both R1 and R2 specimens on day 515 indicated that the SRB colonized the outer layers of the biofilm, either as dense, bright clusters along the edge, or as small spherical groups a little deeper into the granule (Figure 5). By day 304, *Desulfobulbus* spp. occurred sporadically as rod-like clusters (Figure 5A), whereas smaller spheres of Desulfovibrionales detected by the SRB 385 probe were ubiquitous in the sections examined (Figure 5A).

Hybridized sections using the same probes for day 742 on both R1 and R2 showed a similar result. The SRB predominantly colonized the outer edges of the granule. However, at the conclusion of the trial, larger and more abundant clusters of *Desulfobulbus* spp. were observed. In R2 granules, the SRB inhabited the outer layers of the granule, with archaea located closer to the center of the granule (Figure 5C). No SRB were detected in the core of either R1 or R2 granules.

### qPCR analysis

**Figure 6:**
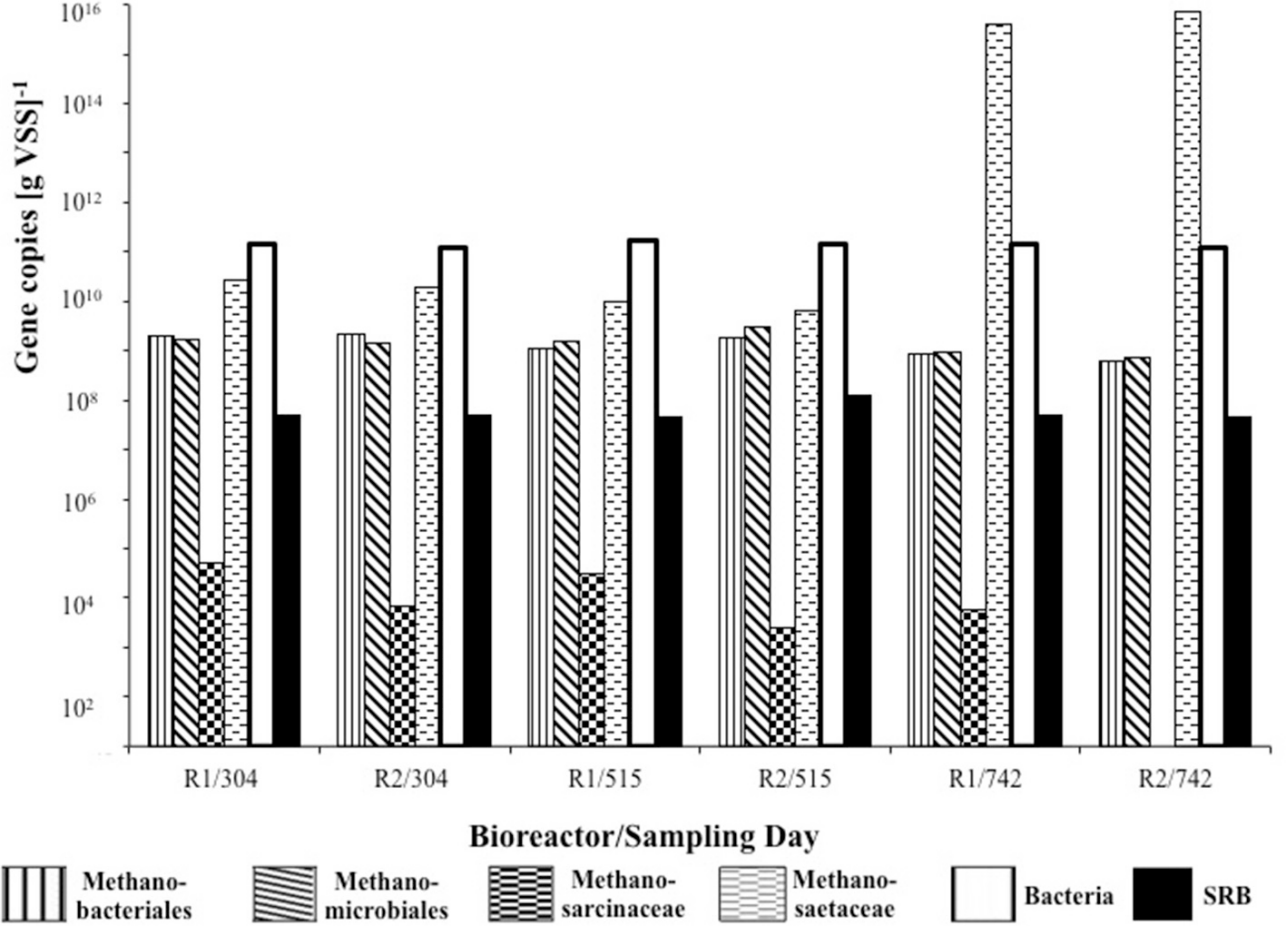
**Quantitative changes in concentration of 16S rRNA genes of bacteria and four methanogenic groups, and in dsrB genes of sulfate-reducing bacteria**.

Of the methanogenic groups analyzed, the Methanosaetaceae were the dominant species in all of the samples from R1 and R2. As the trial progressed, more Methanosaetaceae were detected, whereas the concentration of Methanosarcinaceae genes decreased (Figure 6). For R2, on day 742, no Methanosarcinaceae targets were detected. The concentration of Methanobacteriales and Methanomicrobiales was similar throughout, with only marginally more Methanomicrobiales routinely detected, with the exception of on day 304 (Figure 6). Although sulfate was not present in R1 influent, comparable dsrB concentrations [~10^8^ copies g(VSS)^−1^] were detected in R1 and R2 granules at each of the sampling dates (Figure 6).

## Discussion

### Bioreactor performance

Low-temperature AD trials have previously demonstrated the potential of cold bioreactors for waste conversion (Collins et al., 2003; Enright et al., 2009; McKeown et al., 2012), including for the treatment of acidified, industrial wastewater similar to the feedstock used for this study (Nedwell and Reynolds, 1996; O'Flaherty and Colleran, 1999; O'Flaherty et al., 1999; Fukui et al., 2000). Similarly, successful COD removal (average, >80% efficiency) was achieved by both EGSB bioreactors in this study at 15°C during the start-up phase and throughout the trial. The impact of methanogenic and SRB activity on COD removal, and the interactions between methanogens and SRB, was apparent from bioreactor performance data. COD removal efficiency was not significantly different in R1 and R2, regardless of the presence of sulfate in the influent wastewater. The presence of sulfide indicated dissimilatory sulfate reduction by a sulfidogenic population. Based on the quality of the biogas produced, methanogenesis in the sulfate-amended bioreactor (R2) appeared to only be impacted during the final period (P5) of the bioreactor trial, when the COD: SO^2−^_4_ ratio was 1:2. Even then, the biogas methane concentration was reduced by only 10% compared with the periods before sulfate dosing. Although we do not present volumetric *in situ* methane yield data, and it is possible that methane production was depressed, the biogas quality data shown (Figure 1) indicate that methane concentrations were not diluted by sulfidogenic activity.

O'Flaherty et al. (1998a) and Pender et al. (2004) found that in mesophilic bioreactors treating sulfate-rich wastewaters, all of the methane produced originated from acetate, while H_2_ was consumed by the SRB. These divergent pathways for acetate and hydrogen utilization can facilitate methanogenic and SRB populations to avoid competitive scavenging for available substrates. Moreover, this strategy also avoids impeding the growth of either population. *In situ* COD removal efficiency and biogas methane quality data, however, cannot alone be directly used to address questions on methanogenic-SRB competition, or on sulfide toxicity, in bioreactors. SRB activity can impact methanogenesis directly—through competition for available substrates—or indirectly—through toxicity from by-products, such as hydrogen sulfide. Therefore, the investigations using batch incubations, which were assayed under a range of specific and targeted conditions, are valuable to elucidate interactions along the methanogenic pathway. The assays cannot support differentiation between sulfide inhibition and interspecies competition, as these are largely interdependent i.e., sulfide toxicity in this system will arise from competition provided by the SRB; instead the assays are used to assess the competitive pressure on methanogens from SRB, rather than specific toxicity.

### Impact of sulfate on potential for methanogenic activity

The higher SMAs at 37°C than in 15°C assays was expected, owing to the mesophilic origin of the seed biomass. Generally, due to the scarcity of full-scale, low-temperature anaerobic digesters, the use of biomass from mesophilic AD systems to seed new, cold systems is a likely option in most countries, and was thus the approach taken in this experiment. Although the route of methane production in AD bioreactors is usually through acetoclastic methanogenesis (Scully et al., 2006; Akila and Chandra, 2007; Enright et al., 2009), several previous studies have also found biomass in which hydrogenotrophic methanogenesis was dominant (McHugh et al., 2003; Enright et al., 2005; O'Reilly et al., 2010), as was the case with the seed sludge—and in R1 biomass throughout the trial (Table 4).

The inhibition of hydrogenotrophic methanogenic activity in R1 (control) biomass at 15°C—and in 37°C assays by the conclusion of the trial—indicated the presence of, and competition from, SRB despite the absence of sulfate in R1 influent (Table 4). This was supported by DGGE fingerprinting, qPCR and FISH data (Figures 3, 5, 6).

Indeed, it has been observed that in the absence of sulfate, many SRB ferment organic acids and alcohols, producing hydrogen, acetate, and carbon dioxide, and may even rely on hydrogen- and acetate-scavenging methanogens to convert organic compounds to methane (Plugge et al., 2011). Furthermore, whilst sulfate reducers can also grow without sulfate, in some cases they grow only in syntrophic association with methanogens or other hydrogen-scavengers. Thus, sulfate reducers may compete with methanogens or grow in syntrophy with methanogens depending on the prevailing environmental conditions (Muyzer and Stams, 2008). The dominant methanogens in R1 appeared to be *Methanosaeta*-like organisms (Figure 6), which are acetoclastic and are characterized by high affinity for acetate. Under conditions of low prevailing concentrations of acetate, therefore, *Methanosaeta* will out-compete acetoclastic methanogens with a lower affinity for acetate, such as *Methanosarcina*. SRB may have contributed to the maintenance of a low prevailing acetate concentration in R1, such that the dominant methanogen was *Methanosaeta*.

Conversely, in R2, acetoclastic methanogenesis was the dominant route of methane production, at least at 15°C and at least until day 605 (Table 4). This may be due to a less active hydrogenotrophic methanogenic population owing to competition for H_2_ from SRB, or syntrophic SRB aligning with acetoclastic methanogens resulting in this population shift (Bryant et al., 1967; Schink, 1997; Stams and Plugge, 2009; Plugge et al., 2011). Nonetheless, and interestingly, the assays indicated that sulfate impaired acetoclastic methanogenesis in R2 biomass (Table 4), but the high activity in sulfate-free assays suggests that the toxicity and/or competition was easily reversed, and supports the evidence from DGGE experiments indicating the persistence of acetoclastic methanogens (Figure 3, Table 2).

The findings indicate that acetoclastic methanogenesis was impaired even when the COD: SO^2−^_4_ ratio in the R2 influent was 8:1. Although increased methanogenic activity was observed on acetate in R2 by day 605 (>300 d after SO^2−^_4_ introduction to R2 influent) it was still strongly impaired—up to 69%—by SO^2−^_4_, indicating continued competition from SRB at the lower COD: SO^2−^_4_ ratio at that time.

The data also identify a rather complex situation in R2: SMA against H_2_ in R2 assays was elevated with the addition of SO^2−^_4_. This condition is reflective of R2 *in situ* conditions. This may be due to inhibited homoacetogenic activity, and hence inhibited acetoclastic methanogenic activity, which provides an opportunity for hydrogenotrophic methanogens. For instance, it is widely accepted that H_2_-utilizing SRB out-compete hydrogenotrophic methanogens and homoacetogens because of their lower Km values (higher affinity) (Chaganti et al., 2012). This, in turn, indirectly points to a syntrophic SRB lifestyle in collaboration with hydrogenotrophic methanogens, similar to observations from marine sediments (Plugge et al., 2011).

By the conclusion of the trial, R2 assays (at 15°C) indicated reduced SMA on acetate and increased activity on H_2_, suggesting that the route of methane production had switched to predominantly hydrogenotrophic activity. Despite this, however, the hydrogenotrophic methanogens appeared outcompeted by SRB for H_2_.

Intriguingly, SMA on the indirect substrate, propionate, increased when SO^2−^_4_ was present in assays (at 15 and 37°C), which suggests that propionate oxidation, coupled with SO^2−^_4_ reduction provided methanogenic substrates, which were otherwise unavailable in the absence of sulfidogenesis. Thus, it appears that non-sulfate-reducing propionate-oxidizers—i.e., obligate hydrogen-producing acetogens—were less abundant or less active in R2 biomass.

### Sulfate impacts on community structure and population dynamics, but not on the distribution of SRB, in anaerobic sludge granules

The microbial communities of R1 and R2 diverged during the course of the trial, indicating that the addition of sulfate to R2 influent impacted community structure. Specifically, for example, *Methanosarcina* were undetected in R2 by the final sampling day. However, the physical distribution of microbial groups was not obviously different along the structure of the granular biofilms, with SRB clustering around the surface of sludge granules and with archaea located toward the core of the granules (Figure 5). During the trial, the abundance of dsrB genes was similar in R1 and R2, further indicating a persistent, background population of SRB even in the bioreactor without sulfate addition. Furthermore, little movement was observed in DGGE profile of the dsrB genes. However, the SRB populations detected by FISH experiments appeared to become more abundant in granules over the course of the trial. DGGE profiles and qPCR assays targeting the dsrB mRNA transcripts would provide greater insight; nonetheless, the FISH assays targeting rRNA from SRB do support the conclusion that, although a similar potential for sulfate reduction was present in R1 and R2 biomass, the active portion of the SRB community was more abundant in R2. Microsensor data supported the findings of FISH experiments, indicated an ordered distribution of sulfate reduction and the accumulation of sulfide in the low-temperature granules, as well as indicating the activity of SRB even in the previously unexposed R1 granules.

## Conclusion

COD removal can proceed at 15°C in anaerobic digesters exposed to sulfate. *In situ* methane production appears impacted only at COD: SO^2−^_4_ ratios ≤1:2; thus, higher COD: SO^2−^_4_ ratios would appear to support biogas production in cold anaerobic digesters. Hydrogenotrophic methanogens in low-temperature anaerobic sludge granules were more sensitive to sulfate than acetoclastic methanogens, but complex interactions of SRB, methanogens and homoacetogenic bacteria appear to underpin COD removal by sulfate reduction and methanogenesis.

### Conflict of interest statement

The authors declare that the research was conducted in the absence of any commercial or financial relationships that could be construed as a potential conflict of interest.
